# Repurposing antimalarial aminoquinolines and related compounds for treatment of retinal neovascularization

**DOI:** 10.1371/journal.pone.0202436

**Published:** 2018-09-12

**Authors:** Danielle McAnally, Khandaker Siddiquee, Ahmed Gomaa, Andras Szabo, Stefan Vasile, Patrick R. Maloney, Daniela B. Divlianska, Satyamaheshwar Peddibhotla, Camilo J. Morfa, Paul Hershberger, Rebecca Falter, Robert Williamson, David B. Terry, Rafal Farjo, Anthony B. Pinkerton, Xiaping Qi, Judith Quigley, Michael E. Boulton, Maria B. Grant, Layton H. Smith

**Affiliations:** 1 Cardiovascular Pathobiology Program, Diabetes and Obesity Research Center, Sanford Burnham Prebys Medical Discovery Institute, Orlando, Florida, United States of America; 2 Conrad Prebys Center for Chemical Genomics, Sanford Burnham Prebys Medical Discovery Institute, Orlando, Florida, United States of America; 3 Department of Ophthalmology, Indiana University School of Medicine Indianapolis, Indiana, United States of America; 4 EyeCRO LLC, Oklahoma City, Oklahoma, United States of America; 5 Conrad Prebys Center for Chemical Genomics, Sanford Burnham Prebys Medical Discovery Institute, La Jolla, California, United States of America; 6 Department of Ophthalmology, University of Alabama, Birmingham, Alabama, United States of America; Children's Hospital Boston, UNITED STATES

## Abstract

Neovascularization is the pathological driver of blinding eye diseases such as retinopathy of prematurity, proliferative diabetic retinopathy, and wet age-related macular degeneration. The loss of vision resulting from these diseases significantly impacts the productivity and quality of life of patients, and represents a substantial burden on the health care system. Current standard of care includes biologics that target vascular endothelial growth factor (VEGF), a key mediator of neovascularization. While anti-VGEF therapies have been successful, up to 30% of patients are non-responsive. Therefore, there is a need for new therapeutic targets, and small molecule inhibitors of angiogenesis to complement existing treatments. Apelin and its receptor have recently been shown to play a key role in both developmental and pathological angiogenesis in the eye. Through a cell-based high-throughput screen, we identified 4-aminoquinoline antimalarial drugs as potent selective antagonists of APJ. The prototypical 4-aminoquinoline, amodiaquine was found to be a selective, non-competitive APJ antagonist that inhibited apelin signaling in a concentration-dependent manner. Additionally, amodiaquine suppressed both apelin-and VGEF-induced endothelial tube formation. Intravitreal amodaiquine significantly reduced choroidal neovascularization (CNV) lesion volume in the laser-induced CNV mouse model, and showed no signs of ocular toxicity at the highest doses tested. This work firmly establishes APJ as a novel, chemically tractable therapeutic target for the treatment of ocular neovascularization, and that amodiaquine is a potential candidate for repurposing and further toxicological, and pharmacokinetic evaluation in the clinic.

## Introduction

Two of the leading causes of visual impairment and blindness in the western world are diabetic retinopathy (DR) and exudative age-related macular degeneration (AMD) [1]. Both represent a significant impact on the independence, productivity and quality of life of patients as well as a substantial burden on the health care system. In 2010, more than 6 million Americans suffered from DR and ~2 million suffered from AMD, and the incidence is increasing significantly [2,3,4]. By 2050 the number of Americans with DR and AMD are expected to double [4]. As the lifespan of our population continues to increase, there will be an increasing number of people who are at risk of developing visual impairment. Consequently, the economic burden of visual impairment will continue to grow.

Over the last 15 years, significant advances have been made in the understanding of both DR and AMD. It is now clear that pathological angiogenesis (or neovascularization) contributes to the loss of vision by causing hemorrhage; fibrosis; retinal detachment; and vascular leakage, leading to edema and the deposition of drusden within the retina. Vascular endothelial growth factor (VEGF) plays a key role in this pathophysiology and is the target of current FDA-approved antiangiogenic protein therapeutics [5,6,7,8]. Ranibizumab (Lucentis; Genentech/Roche) [9,10,11], bevacizumab (Avastin; Genentech/Roche) [12,13,14,15] and aflibercept (Eylea; Regeneron Pharmaceuticals)—all anti-VEGF agents—are currently the most common therapies for neovascular AMD. While these therapies have been highly effective, recent studies show a decline in long-term efficacy, which is believed to result from the emergence of VEGF-independent mechanisms and expression of other growth factors and cytokines involved in maintaining the abnormal angiogenic milieu [16,17]. In addition, the further decline in visual function with long-term anti-VEGF therapy has been linked to the loss of the choroidal blood supply, which is in part VEGF-dependent and which supports the integrity and health of the overlying retinal pigment epithelium and neural retina [18,19,20]. Moreover, a significant number of patients receiving anti-VEGF therapies do not benefit and vision continues to diminish [21,22]. The options for these patients are limited. Therefore there is a critical need to target other receptors linked to pathologic neovascularization as an alternative or adjunctive approach to approved anti-VEGF treatments.

Apelin is a peptide hormone recently identified as the endogenous ligand of the APJ receptor [23,24,25] formerly recognized as an orphan G-protein coupled receptor (GPCR). A single gene encodes the pre-pro-apelin protein. Sequential N-terminal deletions produce at least four biologically active apelin peptides: apelin-36, apelin-17, apelin-13 and apelin-12 [26,27]. All apelin peptides are inactivated by removal of the C-terminal phenylalanine residue, catalyzed by the angiotensin converting enzyme 2 (ACE-2) [28]. These four apelin peptides bind and activate APJ [24,29], a GPCR that has been shown to signal via Gαi and ERK pathways [30]. Apelin and APJ are expressed in an array of tissues, and regulate a variety of processes including cardiovascular homeostasis [31,32,33,34,35], food intake [36,37], fluid balance [38,39,40] and cellular proliferation [41,42,43].

While most studies have focused on the cardiovascular effects of apelin, a few have explored its role in both physiological and pathological angiogenesis in the eye [44,45]. Apelin enhances migration, proliferation, and capillary-like tube formation of retinal endothelial cells (RF/6A), but not umbilical vein endothelial cells (HUVEC) [45]. *In vivo*, apelin contributes to retinal vascularization and normal ocular development [46,47], as well as pathological angiogenesis [48,49,50]. Although the precise process by which apelin/APJ promotes retinal angiogenesis is unknown, it is clear that the apelin/APJ system acts synergistically with VEGF via discrete mechanisms to promote vascular development [45,46,47,49,51,52,53,54,55].

Using an existing cell-based assay of APJ signaling [56,57], we screened the Sanford Burnham Prebys (SBP) compound collection of drug-like compounds to identify novel APJ antagonists. We identified multiple compounds that blocked apelin-dependent activation of APJ, including a series of 4-aminoquinolines. The antimalarial compound amodiaquine contains a substituted aminoquinoline core, and was purchased as part of our initial exploration of the structure-activity-relationship between this scaffold and APJ. Amodiaquine antagonized APJ and blocked apelin-induced endothelial tube formation *in vitro*. Further *in vivo* studies in a mouse model of choroidal neovascularization confirmed the antiangiogenic effects of amodiaquine. Mechanistic investigations revealed that amodiaquine acts as a non-competitive inhibitor of apelin-mediated APJ activation. This study, along with a recent report published during the preparation of this manuscript [58], validate small-molecule modulation of APJ as a therapeutic strategy to prevent pathologic angiogenesis in the eye.

## Results

### Identification and validation of aminoquinolines as APJ antagonists

**Fig 1:**
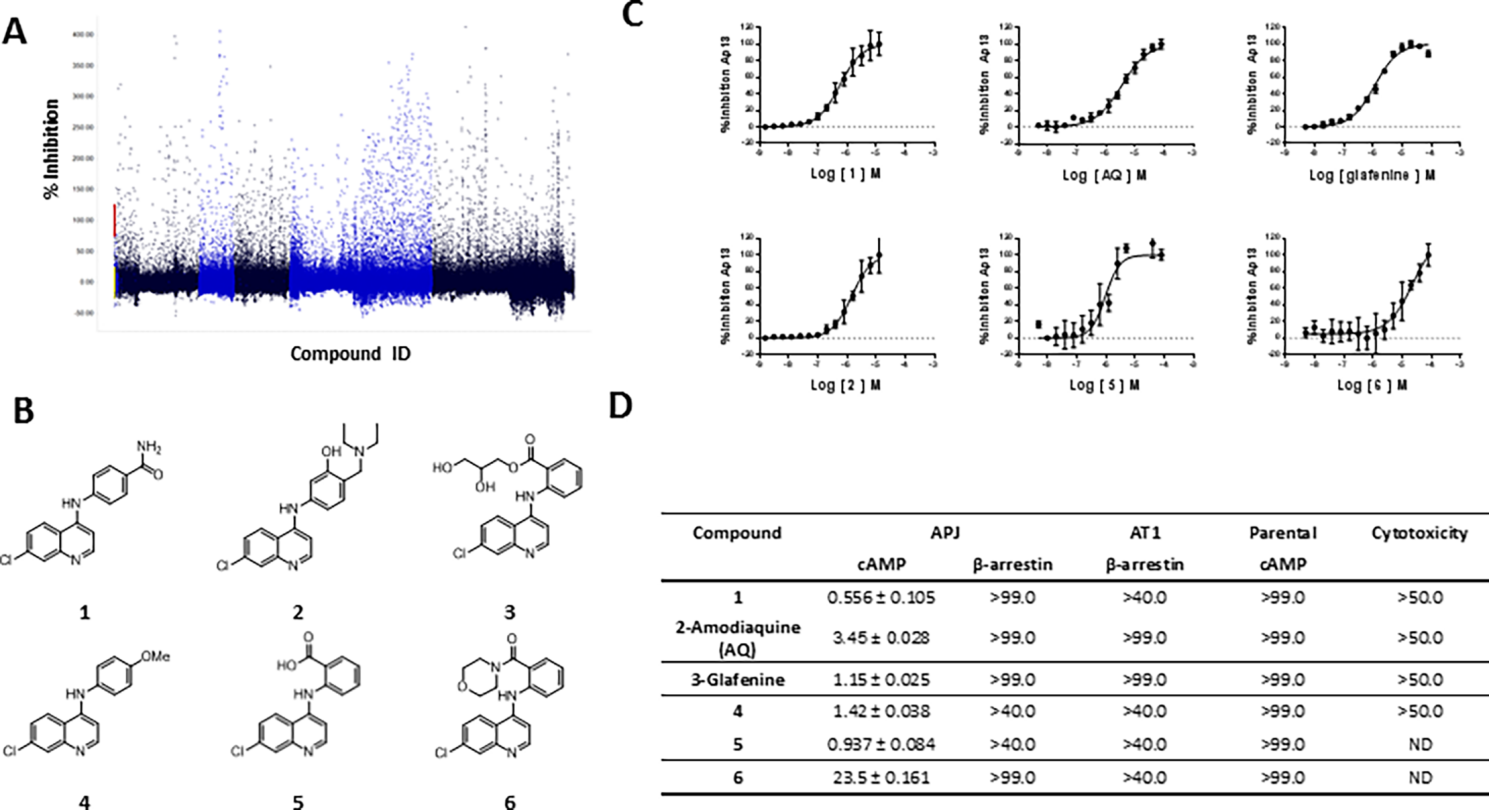
HTS for APJ antagonists and hit confirmation of 4-aminoquinoline analogs. (**A**) Scatter plot of the hits and controls from the HTS. Activity (%) was calculated by normalizing luminescence signal to the mean signal in the dimethyl sulfoxide (DMSO) wells. Compounds were considered hits if they inhibited the response to Ap13 (1 nM) by ≥ 40%. (**B**) Chemical structures of 4-amino-quinolines (4AQs) and (**C**) concentration response curves for hits and analogs. The percent inhibition of 1nM Ap13 is shown. The inset table (**D**) shows the IC_50_s of the 4AQs in the primary APJ assay (cAMP), secondary assay (APJ β-arrestin recruitment) and counter assays (AT1 β-arrestin recruitment, and parental cells (cells lacking APJ cAMP). General cytotoxicity was assessed using ATP-lite assay and Fa2-N4 cells as described. All IC_50_ data are reported in μM. Data are means ± SEM (*n* = 3). Curves represent the best fit non-linear regression analysis calculated using a 4-paramter logistic with GraphPad Prism7.

To identify small-molecule antagonists of the apelin receptor (APJ) we interrogated the SBP compound file of ~425,000 compounds using CHO-k1-AGTRL1 cells overexpressing human APJ and a competitive immunoassay of intracellular cAMP [59]. The compound file was screened at a concentration of 10 μM. For all 333 plates assayed, the average Z-factor = 0.68 and the average S/B = 8.2, indicating a robust assay performance. Active compounds were those that inhibited Ap13 (1.0 nM)-mediated decrease in forskolin stimulated intracellular cAMP by ≥ 50%. There were 2550 compounds that met this criterion, representing a hit rate of ~0.6%. **Fig 1A** shows a scatter plot of the screening data. Included in this hit set was a series of aminoquinolines, exemplified by compound **1** (**Fig 1B**). A substructure similarity search of the SciFinder database revealed that **1** shared a common 4-chloro-aminoquinoline core with two approved drugs, amodiaquine (**2,** hereafter **AQ**) and glafenine (**3**). As part of an initial hit-validation strategy, these compounds and three other commercially available analogs were purchased for further testing. The purity and identify of all compounds were confirmed by NMR and LC-MS, and they were subsequently retested in a battery of cell-based assays of APJ function and counter screens. When tested in the primary APJ cAMP assay, all six aminoquinolines were found to be potent APJ antagonists (**Fig 1C and 1D**). When tested in cells lacking the receptor (parental CHO-K1) or in those expressing the closely related angiotensin II type 1 (**AT1**) [60], all six compounds demonstrated selectivity for APJ. Interestingly, neither AQ nor **1, 3, 4, 5, or 6** antagonized Ap13-mediated recruitment of β-arrestin suggesting a selective, or biased, antagonism [61] of G-protein-dependent signaling by APJ. None of the compounds exhibited cytotoxicity when tested on Fa2-N4 hepatocytes at concentrations up to 50 μM. (**Fig 1D**). Compared to the aminoquinoline compounds shown in Fig 1, AQ was chosen for potential repurposing for the following reasons: 1) AQ is often used as both a prophylactic and a treatment for *Plasmodium falciparum* infection and has fewer, less severe side effects than **3** (glafenine); 2) although some analogs were more potent, these compounds have not been used in humans and thus were not suitable for a repurposing strategy, and 3) the potential for further structural modification to increase APJ antagonism and reduce side-effects.

**Fig 2:**
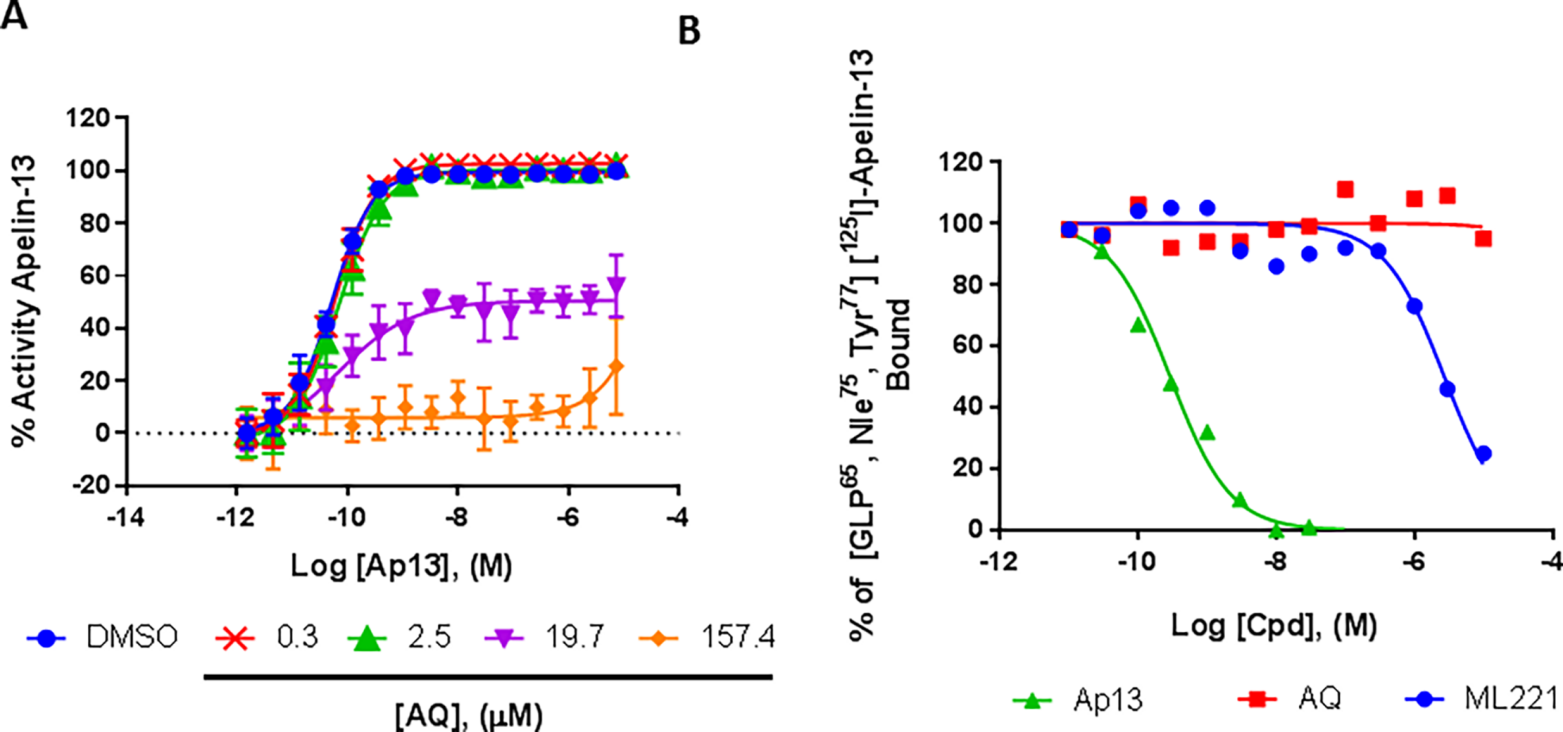
AQ is a non-competitive apelin receptor antagonist. (**A**) Ap13 concentration response curves showing the effect of pre-incubation either in the absence (○) or with different concentrations of AQ for 0.5h at 37°C: 0.3 μM (red ×), 2.4 μM (green △), 19.7 μM (purple ▼), 157.4 μM (orange ◆), after which increasing concentrations of Ap13 were added and the incubation continued for 0.5h. AQ reduced the E_max_ of Ap13 responses, reflecting insurmountable inhibition. Data are mean ± SEM (*n* = 3). (**B**) Radioligand binding inhibition curves showing percent bound [^125^I]-Glp65, Nle75, Tyr77-Ap13 with different concentrations of cold, unlabeled Ap13 (green △), and the competitive apelin receptor antagonist ML221 (blue •) and AQ (red ■). Data shown are the mean ± SEM of two independent experiments (*n* = 2) with each data point performed in duplicate. All curves overlaying the data points represent the best fit line of non-linear regression analysis performed as described in Materials and Methods.

To better characterize the mechanism by which AQ antagonizes APJ, and determine the potency of the antagonism, we next tested the ability of AQ to decrease the EC_50_ of Ap13. AQ failed to produce parallel shifts in the concentration response curves for Ap13. At concentrations above 2.4 μM, AQ shifted the EC_50_ of Ap13 and reduced E_max_ correspondingly (**Fig 2A**). At higher concentrations, AQ virtually eliminated all response to Ap13. The pA2 could not be calculated because the assumptions of the Schild analysis were not met [62]. Consistent with these functional results, AQ did not displace [^125^I]-Glp65, Nle75, Tyr7-Ap13 binding to membranes containing APJ when tested as high as 100 μM. In contrast, unlabeled Ap13 and the prototypical APJ antagonist ML221 [57] effectively displaced [^125^I]-Glp65, Nle75, Tyr7-Ap13 with K_i_ = 0.18 nM and 1.33 μM respectively (**Fig 2B**). Taken together, these data indicate that AQ is a non-competitive APJ antagonist.

### Anti-angiogenic activities of APJ antagonists

**Fig 3:**
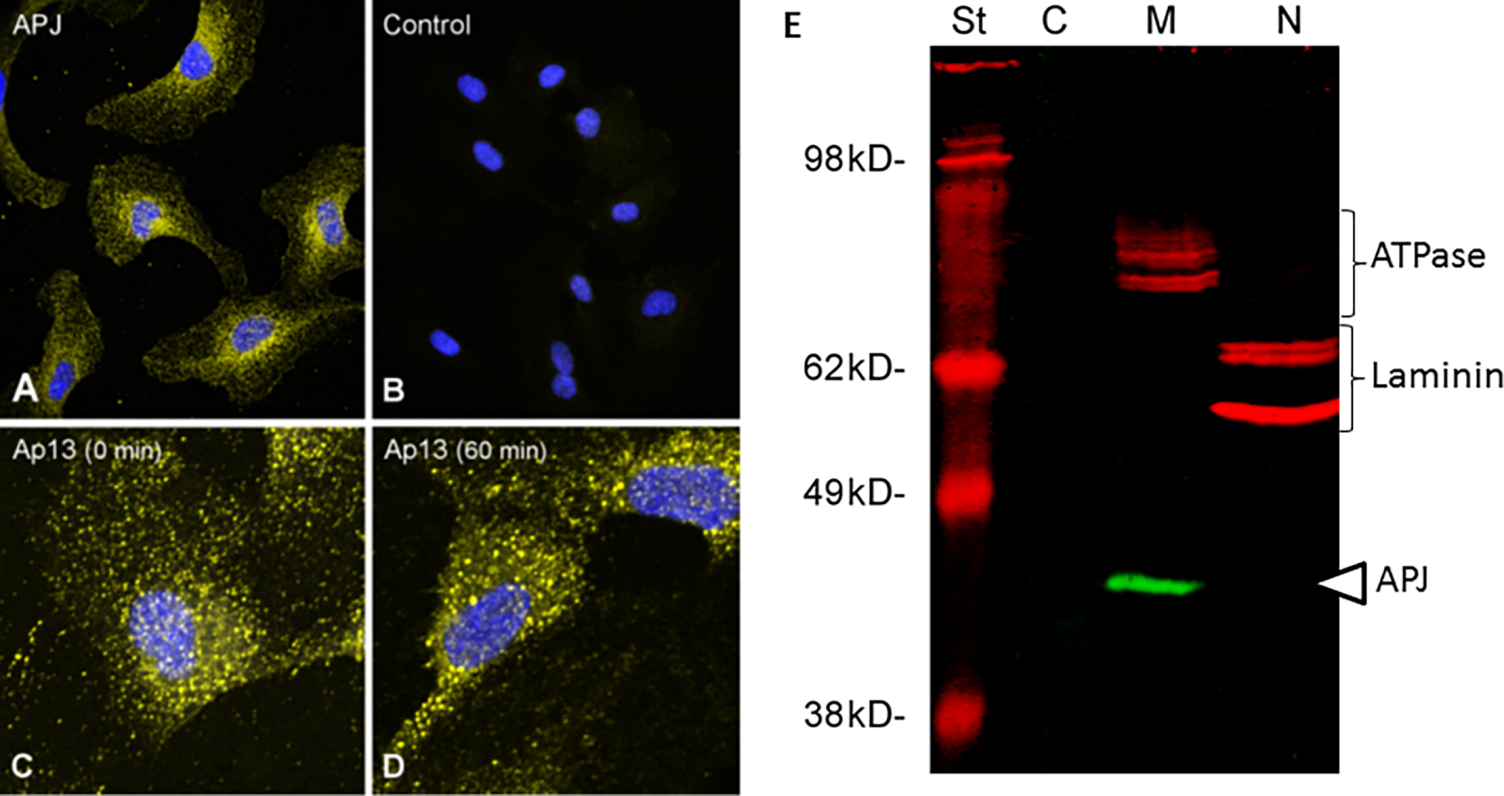
Human retinal endothelial cells (HREC) express APJ. (**A**) APJ protein was detected and visualized in HRECs by immunocytochemistry using the anti-APJ antibody ab140508, and Alexa488 conjugated secondary antibody. (**B**) A control experiment in which the primary anti-APJ antibody was omitted shows the specificity of APJ immunoreactivity. (**C, D**) Incubation of HRECs with Ap13 (1 nM) led to the aggregation of APJ immunoreactivity into bright puncta after 60 min. Cell images are maximal intensity projections taken using a confocal microscope and psuedocolored for ease of interpretation. APJ is shown in yellow or white. Nuclei were visualized using DAPI and colored blue. (**E**) Cytosolic (c), membrane (m) and nuclear (n) fractions of HREC cells were isolated and subjected to SDS-PAGE and Western blotting as described. APJ (green band indicated by a white triangle on the right of the image) immunoreactivity was observed only in the membrane fraction with a migration of < 49 kDa. The efficiency of the cellular fractionation was monitored by blotting for the membrane bound Na/KATPase (top bracket on right) and nuclear laminin (bottom bracket on right), both shown in red. St = molecular weight marker.

We next sought to determine the effect of AQ on the proliferation and migration of human retinal microvascular endothelial cells (HRECs). Previous studies have reported that endothelial cells derived from multiple distinct vascular beds express APJ and respond to Ap13 [45,49,63,64,65]. However, there are no published studies in which APJ expression has been confirmed in HRECs. Therefore, we applied immunohistologic and western blotting approaches to confirm endogenous expression of APJ in these cells. A polyclonal antibody targeting the intracellular C-terminal tail of human APJ and a fluorescently conjugated secondary detection antibody, identified APJ immunoreactivity throughout the cell in a generally diffuse speckled pattern (**Fig 3A**). APJ immunoreactivity was not evenly distributed; it appeared to be enriched around the nucleus. This is likely reflective of the morphology of the HREC where the nucleus constitutes the thickest part of the cell. This staining pattern is consistent with that previously reported for APJ in HUVECs [65], rhesus choroid endothelial cells [49] and retinal pericytes [66], as well as other GPCRs [67]. APJ immunoreactivity in HRECs was specific (**Fig 3B)**, and was confirmed using three additional APJ antibodies targeting other epitopes within the protein, and qPCR (Figure A and Table A in S1 File). The cytosolic, nuclear and membrane fractions of HRECs were subjected to western blotting using the same antibody. APJ immunoreactivity was highly enriched in the membrane fraction, but not the cytosolic or nuclear fractions. A single band of slightly less than 49 kD was observed. This molecular weight is consistent with a predicted APJ molecular weight of ~43 kD. The efficiency of the cellular fractionation method was monitored by blotting for the membrane bound Na/K ATPase and nuclear laminin (**Fig 3C**). When HRECs were incubated with Ap13 (1.0 nM) for 60 min, the pattern of APJ immunoreactivity shifted from a diffuse speckled pattern to one characterized by aggregated bright puncta (**Fig 3D and 3E**), indicating that the addition of ligand coalesced APJ immunoreactivity into pits characteristic of activated GPCRs [68,69].

**Fig 4:**
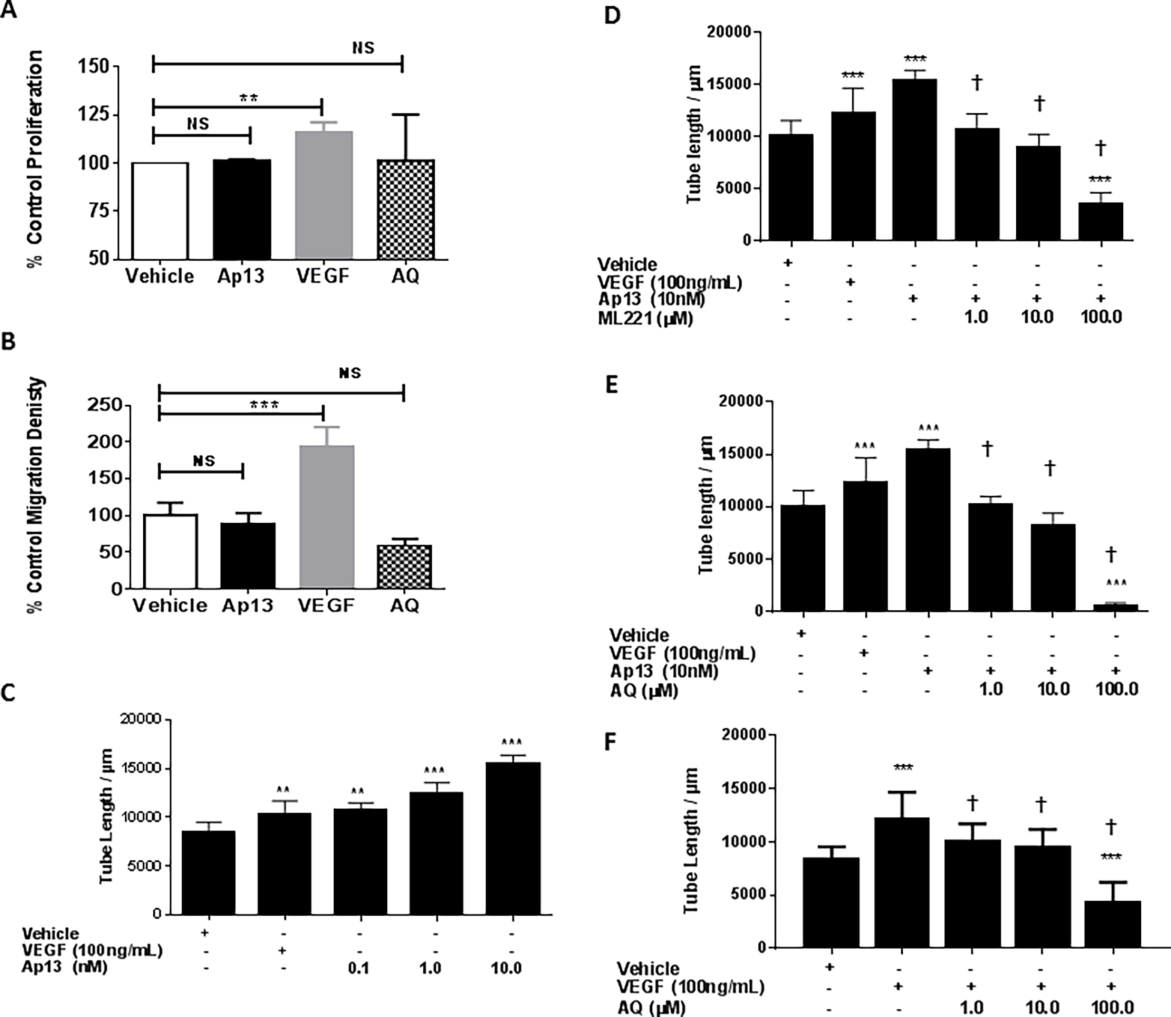
Effects of Ap13 on HREC proliferation, migration and tube formation. (**A**) Proliferation: human retinal endothelial cells (HRECs) were exposed to vehicle (DMSO 1% v/v), VEGF (100 ng/mL), and Ap13 (10 nM) for 16 h. Proliferation was determined as described in Material and Methods. (**B**) Migration. Data plotted is the mean percent (%) change ± SEM normalized to vehicle control. NS = not significant; ** = p<0.01; *** = p<0.001 vs vehicle; as determined by ANOVA with Dunnett’s test for comparison to control.(**C**) Ap13 induces HREC tubular network formation *in vitro*. (**D, E**) The prototypical APJ antagonists ML221 and AQ block Ap13-induced HREC tube formation in a concentration dependent manner. (**F**) AQ blocks VEGF-induced HREC tube formation in a concentration dependent manner. Data plotted is the mean ± SEM length of endothelial tubes measured in micrometers (μm), normalized to vehicle control. Mean and SEM are calculated from an experiment that was performed twice with each treatment condition tested in triplicate (*n* = 3). NS = not significant; ** = p<0.01; *** = p<0.001 vs vehicle; † = p<0.0001 compared to cells exposed to VEGF alone (100 ng/mL) as determined by ANOVA with Tukey’s multiple comparison test.

Having confirmed that HREC cells express APJ, we next evaluated the effect of Ap13 and its inhibitors on HREC proliferation, migration and tube formation. As expected, the proangiogenic VEGF (100 ng/mL) stimulated HREC proliferation and migration (**Fig 4A and 4B**). In contrast, Ap13 had no significant effect on either proliferation or migration of HRECs (**Fig 4A and 4B**). Both VEGF and Ap13 stimulated the formation of endothelial tubes. When tested at multiple concentrations, Ap13 increased overall tube length in a concentration-dependent manner that was equivalent or greater than VEGF (**Fig 4C**). The combination of Ap13 with VEGF did not significantly increase the extent of tube formation formed when either factor was added alone, indicating that Ap13 and VEGF do not act synergistically on HRECs (Figure B in S1 File).

Both AQ and the prototypical APJ antagonist M221 blocked Ap13-dependent increases in tube formation. For both compounds this effect was concentration-dependent (**Fig 4D and 4E**). Of In order to clarify the relationship between apelin and VEGF in this system, and to test the hypothesis that AQ antagonizes HREC tube formation independent of VEGF signaling, we examined the effect of AQ on VEGF-induced tube formation. In the absence of exogenous Ap13, AQ blocked VEGF-induced tube formation (**Fig 4F**). A similar effect was observed for ML221 (Figure C in S1 File), indicating that the opposition to VEGF-induced tube formation was mediated by the selective antagonism of APJ by AQ and ML221. This effect was sufficiently pronounced that it decreased the overall tube length compared to vehicle. We performed a cell viability assay on HRECs to ensure that this observation was not reflective of cytotoxicity. Neither ML221 nor AQ were significantly toxic to HRECs (**Table 1**) when tested up to 100 μM.

**Table 1:** Profile of AQ absorption, distribution, metabolism, elimination and toxicity (ADME/T).

Compound			AQ (2)
**Polar surface area** (A^2^, calculated by ChemBioDraw)			47.86
**CLogP**			5.46
**LogD** (pH 7.4)			2.37
**Aqueous solubility** (μg/mL) pH 5.0 / 6.2 / 7.4			>35 / > 48 / 17
**PAMPA permeability** Pe (x10^-6^ cm/s) Donor pH: 5.0/ 6.2/7.4; Acceptor pH: 7.4			3.0 / 9.6 / 54.0
**Plasma-protein binding** (% bound)	Human 1 μM / 10 μM		88.2 / 83.8
	Mouse 1 μM / 10 μM		81.9 / 70.8
	Rat 1 μM / 10 μM		75.3 / 76.8
**Plasma stability** (% remaining at 3 hrs) Human / Mouse / Rat			37.3 / 36.1 / 46.3
**Hepatic microsome stability** T1/2 (min) Human / Mouse / Rat			17.0* / 39.8 / 38.9
**Cytotoxicity** LC_50_ (μM)	Fa2N-4 cells		> 50
	HRECs		> 100

### *In vitro* ADME and tissue distribution of amodiaquine

To determine the suitability of the compound for use in *in vivo* efficacy studies, the ADME/T (absorption, distribution, metabolism, excretion, and toxicity) and pharmacokinetic properties of AQ were evaluated (**Table 1**). At physiological pH (7.4), the LogD of AQ is optimal. Accordingly, AQ was moderately soluble in aqueous media and exhibited moderate permeability in the PAMPA assay. Plasma protein binding (<90% bound) and stability (>25% remaining at t = 3 h) were within the acceptable range [70,71]. In human microsomes, AQ exhibited a two-phase exponential decay with a terminal half-life of 17.0 min. In rats and mouse hepatic microsomes, AQ exhibited a slower rate of decay best described as a single phase with a half-life of 38.9 and 39.8 min respectively (Figure D in S1 File). The primary metabolite of AQ is a desethyl form resulting from CYP450 metabolism [72]. Thus it is not surprising that AQ was rapidly metabolized by human, mouse and rat liver microsomes. Indeed, the metabolism of AQ coincided with the appearance of the desethylamodiaquine (DEAQ) in both human and mouse, but not in rat microsomes. Regardless, DEAQ was an effective APJ antagonist equipotent to the parent AQ (Figure E in S1 File). When tested on hepatocytes, AQ showed no signs of cytotoxicity at up to 50μM. Similarly, AQ was not cytotoxic to HRECs at up to 100 μM.

The distribution of AQ in the mouse eye was evaluated after a single intravitreal dose of 50 μg. **Table 2** shows that after 24 h AQ is found in the target tissue, the RPE/choroid/sclera section of mouse eyes receiving compound at levels that exceed the cellular EC_50_ by ~20 fold. The retina contained a modest amount of compound, while the vitreous contained less than was accurately quantifiable via LC/MS. The compound was persistent showing sustained levels in the target tissues 7 d after administration. No detectable levels of AQ were observed in the plasma of mice after 7 d IVT administration.

**Table 2:** Distribution of AQ in the mouse eye after intravitreal injection.

Tissue	AQ (ng)		Ratio Tissue mass / Cellular potency	
	24h	168h	24h	168h
**Vitreous**	BLOQ	BLOQ	ND	ND
**Retina**	40.0 ± 0.8	45.0 ± 0.9	5	9
**RPE/Choroid/Sclera**	161.0 ± 2.5	215.0 ± 7.1	20	27

### AQ decreases pathological neovascularization *in vivo*

**Fig 5:**
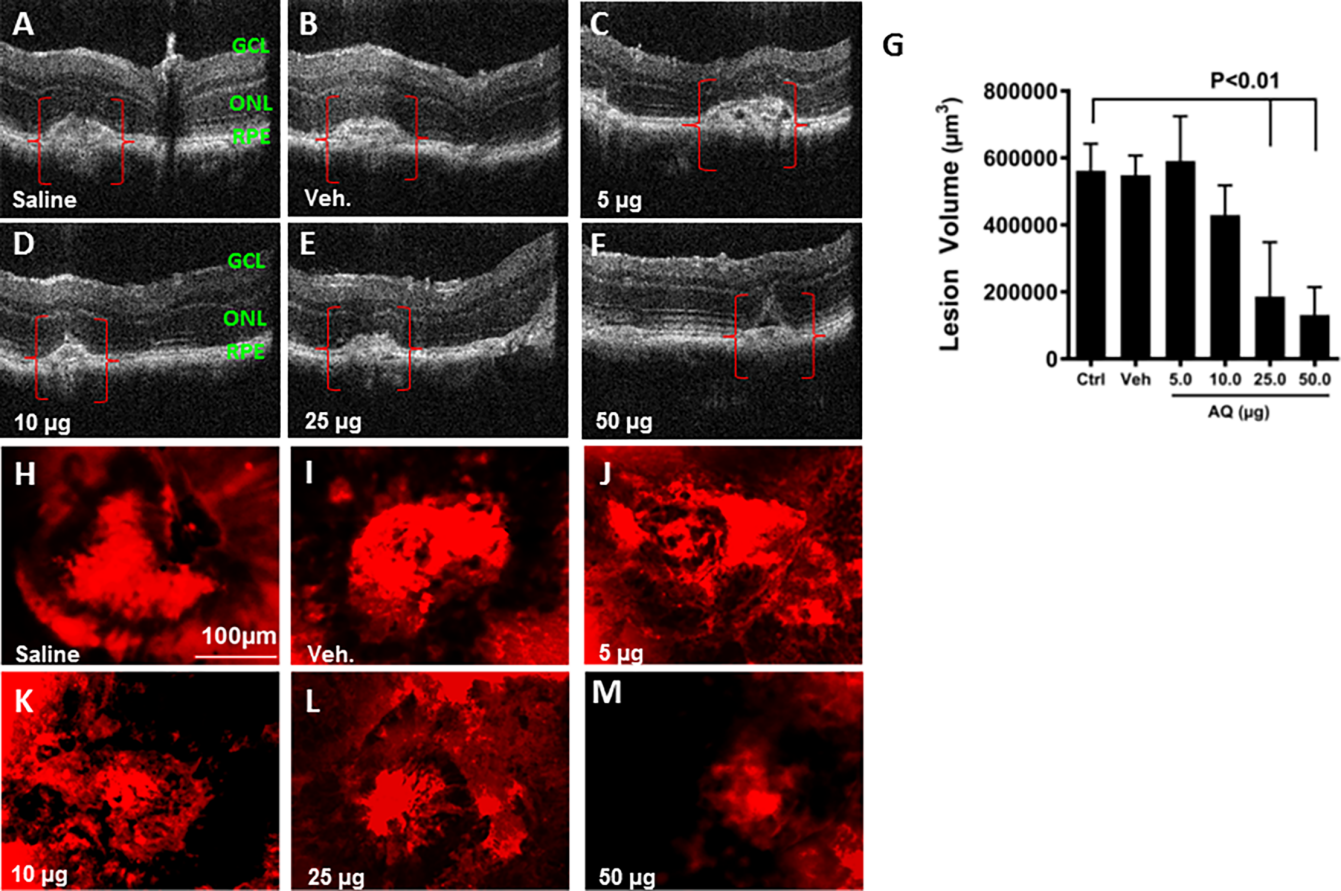
*In vivo* efficacy of amodiaquine in mice with CNV. (**A-F**) Optical coherence tomography (OCT). Representative images of laser-induced CNV lesions (red brackets), showing a significant reduction in size and/or in intensities (black holes) of injury after the treatment with AQ. (**H-M**) RPE choroidal flat mounts were stained with agglutinin-TRITC conjugate (*red*) to visualize the CNV lesions by confocal microscopy. Saline treated eyes (**A, H**). Vehicle (1% DMSO) treated eyes (**B, I**). AQ treated eyes (**C-F, and J-M**). AQ doses administered via intravitreal injection are indicated in each panel. Note the reduction in lesion size and the spotted black replacement to the red staining in eyes treated with AQ, indicative of angiogenesis subsiding which showed most effectively at the dose of 50 μg. (**G**) Quantification of the CNV lesion volume from Z-stack images of choroidal flatmounts stained with agglutinin was made using ImageJ software, and demonstrate a significant reduction in CNV lesion volume after AQ intravitreal injection compared to vehicle-treated controls. P>0.01. Mean ± SEM, n = 5. SEM, standard error of the mean. Statistical significance was determined using ANOVA with Tukey’s multiple comparison’s test.

Despite poor microsomal stability, the combination of balanced solubility and permeability, low protein binding, low cytotoxicity and an equipotent active metabolite led us to advance AQ into *in vivo* efficacy studies. Wild type C57BL/6J mice were subjected to laser-mediated disruption of Bruch’s membrane to induce choroidal neovascularization. One day after laser injury, AQ was administered via intravitreal injection at 5.0, 10.0, 25.0, or 50.0 μg. To ensure adequate ocular exposure to AQ, a second injection at the same dose followed six days later (seven days post injury). The CNV lesion size was significantly smaller in in the AQ treated eyes than those in vehicle treated eyes 14 days post-laser injury, as monitored *in vivo* by optical coherence tomography (OCT) (**Fig 5A–5F**). These decreases are comparable to those we have previously obtained using an anti-VEGF164 antibody, which is a murine-optimized equivalent of bevacizumab, the standard of care in humans [73,74]. Confocal images of agglutinin-stained choroidal flatmounts revealed a reduction in CNV lesion size at 10μg AQ and higher (**Fig 5H–5M**). Although there was not a statistically significant reduction in the CNV lesion volume compared to the vehicle control in eyes treated with AQ at 5.0 and 10.0μg, there was a statistically significant decrease in CNV lesion volume at 25.0μg and an even greater decrease at 50.0 μg compared to the control eyes (**Fig 5G**). Consistent with the *in vitro* cytotoxicity assays, there were no signs of toxicity to the ocular tissues in the eyes of mice injected with AQ.

## Discussion

The apelin receptor represents a novel drug target for the treatment of ophthalmic diseases characterized by neovascularization like DR and AMD. *In vitro*, apelin stimulates endothelial cells to proliferate, migrate and to form a tubular network. In preclinical models including zebrafish and mice, apelin has been shown to play a critical role in the development of the retinal vasculature *in vivo*. Importantly, these effects were shown to be independent of VEGF signaling. Consistent with these findings mice genetically deficient in APJ were observed to be protected from laser-induced CNV [48]. This compelling evidence led us to hypothesize that small molecule antagonists of APJ would be an effective therapy for patients who are refractory or non-responders to current standard of care.

Here we report the discovery that 4-chloro-aminoquinolines, typified by the antimalarial drug amodiaquine and related compounds are potent and selective antagonists of APJ. As shown above, a series of related analogs including the NSAID glafenine exerted similar antagonist effects on APJ. This effect appeared biased, blocking only APJ-dependent cAMP signaling, while having no effect on β-arrestin recruitment. When administered intravitreally, amodiaquine penetrated the subretinal layers of the anterior eye where it effectively decreased the size and extent of laser-induced CNV. Despite being rapidly metabolized by CYP450s, amodiaquine achieved persistently high concentrations in the target tissue sufficient to elicit the therapeutic effect. At its highest dose, amodiaquine virtually eliminated the neovascular lesion.

AQ is a non-competitive antagonist of APJ signaling. Whether related 4-cholor-aminoquinlines have the same pharmacology remains to be determined, but the limited data set obtained suggests that APJ antagonism may be a class effect. Indeed, all six related compounds tested share the fundamental 4-cholro-amino-quinoline core, and act as potent, fully efficacious APJ antagonists. A more thorough evaluation of the pharmacology, and structure-activity-relationships of these analogs is necessary to confirm this hypothesis. Such work is beyond the scope of this report, but the facile chemistry, established syntheses, and number of areas of the compound available for substitution, suggest that can be readily conceptualized and handily executed. Further exploration and exploitation of the SAR may also yield novel chemical entities with improved potency, and pharmacological properties.

Our results demonstrate that APJ antagonism appears to block angiogenesis induced by both Ap13 and VEGF, when applied alone and in combination. Based on the data obtained, it does not appear that antagonism of APJ disrupts a synergistic interaction between APJ and VEGF signaling. In consideration of the key role played by apelin in the remodeling of extracellular matrix remodeling [31], and its localized expression to endothelial tip cells [75], it is tempting to hypothesize that the antagonism of APJ prevents angiogenesis by opposing the effects of Ap13 on matrix remodeling. Indeed, we have previously shown that Ap13 suppresses the expression of the matrix forming genes plasminogen activator inhibitor-1 (PAI-1), collagen 1a1, tissue inhibitor of matrix-metalloprotinease-1 (TIMP-1), fibronectin and integrin-β, while simultaneously stimulating expression of the matrix degrading enzymes of matrix-metalloprotinease-2 (MMP-2) [31]. Under normal development and pathological conditions, the formation and maturation of new blood vessels requires an extracellular matrix environment conducive to the proliferation and migration of endothelial cells [76]. Blocking the effect of Ap13 on matrix remodeling could effectively deny the activated endothelial cell the opportunity to proliferate and migrate, thus mitigating the overall proangiogenic effect of both VEGF and Ap13. Additional studies are required to validate this hypothesis.

AQ is a 4-aminoquinoline that is widely used for both prophylaxis and treatment of malaria [77,78]. This class of antimalarial drugs possess side effects and toxicities ranging from the life threatening to the mundane [79,80,81]. In the US, amodiaquine was withdrawn from the market because of hepatitis and agranulocytosis [82]. After oral administration, amodiaquine is rapidly metabolized to DEAQ, by the cytochrome P450 enzyme 2C8 [80]. It is this metabolite that is thought to be responsible for these rare, but fatal side effects. Additionally, there are well documented ophthalmologic reactions associated with use of aminoquinolines that must be weighed when considering the use of the compound for CNV. Of these retinopathy is rare, but the most serious [83]. Needless to say, any repurposing of AQ must address the hematologic, hepatic and ophthalmic side effects. Although in our study, amodiaquine persisted in the eye up to seven days post injection, the level of systemic exposure was below the limit of quantitation. This suggests that intravitreal administration of the drug has the potential to ensure delivery to the desired target tissue while limiting systemic exposures that lead to these toxic effects. However, it remains to be seen if the mild, reversible blurring of vision known to occur with long term exposure to amodiaquine, or the more profound retinal toxicities will occur when administered IVT at an effective dose in humans. Further preclinical studies are necessary to thoroughly assess the potential risk of impaired vision and retinopathies before clinical studies are conceived. It is worth nothing that there were no signs of overt systemic or ophthalmic toxicity in either the efficacy study or the tissue distribution studies that were executed as part of this study.

In summary, this study validates targeting APJ for the treatment of CNV and provides compelling evidence that AQ, a widely used anti-malarial drug, has a strong anti-angiogenic activity *in vitro* and *in vivo* in a mouse model of AMD. We believe these results provide sufficient evidence to support clinical investigations into the use of amodiaquine alone and in combination with other anti-VEGF drugs. The repurposing or repositioning of AQ in AMD treatment may provide a new therapeutic avenue for patients who fail to respond to existing anti-VGEF therapies. Moreover, as a small molecule, free from composition of matter patent limitations, AQ is a significantly less expensive alternative to the current costly options. This study also lays the foundation for further structure modification of 4-aminoquinolines to discover more optimal analogs for the treatment of CNV.

## Materials and methods

### Peptides and compounds

Ap13 peptides were purchased from 21^st^ Century Biochemicals, Inc (Marlborough, MA). [^125^I]-Glp65, Nle75, Tyr77-Ap13 was purchased from Perkin Elmer (Waltham, MA). Compounds AQ and **3** were from Spectrum Chemical. Compounds **1, 4–6** were synthesized in house (see **Figure D in S1 File** for synthetic scheme). All other chemicals and reagents were from Sigma-Aldrich (St Louis, MO) except **ML221** which was prepared as described previously [57,84]. The identity and purity of all compounds were confirmed using LC-MS and NMR.

### Animal studies

All mice were housed under specific-pathogen-free conditions and handled in accordance with the ARVO statement for Use of Animals in Ophthalmic and Vision Research and the guidelines of the Institutional Animal Care and Use Committee at University of Alabama, Birmingham, EyeCRO, and Sanford Burnham Prebys Medical Discovery Institute at Lake Nona. The animal experiments performed in this study followed protocols that were specifically approved by the Institutional Animal Care and Use Committee of SBP at Lake Nona, The University of Alabama at Birmingham and EyeCRO (Protocol 2017–0160, approved 01/29/2018) was in accordance with AVMA and ARVO guidelines.

### Laser-induced CNV model

The laser procedure was undertaken as previously described [85,86]. Briefly, 8-week-old female C57BL/6J mice (n = 5/treatment group) were anesthetized with a mixture of ketamine (80 mg/kg) and xylazine (10 mg/kg) and their pupils dilated with tropicamide (0.5%) and phenylephrine (2.5%). Under a fundus microscope an argon green ophthalmic laser, coupled to a slit lamp set to deliver a 50 ms pulse at 200mW with a 50 um spot size, was used to rupture Bruch’s membrane in three quadrants of the right eye located approximately 50 mm from the optic disc at relative positions of 9, 12 and 3 o’clock. The left eye served as an untreated control. Mice were assessed using optical coherence tomography and euthanized 14 days after laser injury. At the time of euthanasia, the eyes were collected, enucleated for histological analysis and fluorescent staining.

### Treatment regime

Mice received intravitreal injection (1.0 μl/eye/injection) into the right eye while the left eye acted as the uninjected control. Animals were randomly assigned to one of six treatment groups each consisting of n = 5 mice: saline or vehicle (DMSO), or compounds (0.0–50.0 μg). On the day of injection the compounds were formulated in 100% DMSO and administered to mice via intravitreal injection immediately after laser injury and again 7 days after laser injury. This second injection ensured sufficient compound would be available for the duration of the experiment.

### Optical coherence tomography (OCT)

OCT was performed at the indicated times using the Micron III intraocular imaging system (Phoenix Research Labs, Pleasanton, CA, USA). Before the procedure, eyes were dilated with 1% tropicamide solution (Alcon, Fort Worth, TX, USA) and lubricated with hypromellose ophthalmic demulcent solution (Gonak) (Akorn, Lake Forest, IL, USA). Mice were then placed on a custom heated stage that moves freely to position the mouse eye for imaging. Several horizontal and vertical images were taken per lesion. In addition, gross retinal/choroidal structure, and vascular patterns were examined for possible adverse effects of the test compound or vehicle.

### Measurement of lesion volume *ex vivo*

For measuring lesion volume we used a vascular specific dye, Ricinus Communis Agglutinin I, conjugated to rhodamine (Vector Laboratories, Inc.), to label whole flat mounts of RPE/choroid/sclera which were incubated for 30 minutes at room temperature in 1:400 of 10 mM HEPES, 150 mM NaCl and 0.1% Tween 20. The tissues were covered in aqueous mounting medium (VectaShield; Vector Laboratories, Inc.) for observation on a confocal microscope (Olympus DSU-Olympus IX81; Olympus America, Inc., Center Valley, PA). Digital images were captured by using imaging software (SlideBook 4.2; Intelligent Imaging Innovations, Inc., Denver, CO) in a three-dimensional stacked manner to facilitate volumetric analysis from experimental and control samples with identical photomultiplier tube gain settings. The confocal images were then processed identically in experimental and control eyes and measured using ImageJ software. In all CNV studies, animals were randomized and treatments blinded until all analysis is complete. All determinations were performed in at least 5 mice/group.

### Tissue distribution study

The distribution of AQ into the vitreous humor, retina, and combined RPE/choroid/sclera of the eyes of mice receiving AQ was evaluated 1 day and 7 days after intravitreal injection. Mice were assigned to groups (n = 6/group) that received either 50 μg compound, or vehicle via intravitreal injection (1 μl volume). One day after injection, ½ of the mice (*n* = 3 / group) mice were euthanized. The eyes were enucleated and the vitreous humor, retina, and RPE/Choroid/sclera were isolated and snap frozen in liquid N2. The remaining mice in each treatment group (*n* = 3 / group) were euthanized and the same tissues collected. The quantity of compound in the tissues (*n* = 6 eyes / treatment group) was measured using LC-MS/MS.

### Cell culture

PathHunter™ GPCR Arrestin and cAMP Hunter™ cell lines (DiscoveRx Corp., Fremont, CA) were used to assay G-protein-dependent signaling and β-arrestin recruitment to APJ. CHO-K1 cells stably expressing APJ (CHO-K1-APJ) or AT1 (CHO-K1-AT1) with β-arrestin/β-galactosidase enzyme fragment complementation constructs were maintained in HAM's F-12 medium (Hyclone, Logan, UT) supplemented with 10% FBS, 1X Penicillin-Streptomycin-Glutamine (Invitrogen; Carlsbad, CA), 300 μg/ml hygromycin (EMD Biosciences, San Diego, CA), and 800 μg/ml Geneticin (Cellgro, Manassas, VA). Primary Human Retinal Microvascular Endothelial Cells (HRECs) were purchased from Cell Systems (Kirkland, WA) and maintained in Complete Classic Medium with CultureBoost-R™ (Cell Systems). Parent CHO-K1 cells were obtained from ATCC and maintained in HAM’s F-12 medium supplemented with 10% FBS, and1X Penicillin-Streptomycin-Glutamine (Invitrogen; Carlsbad, CA). All cells were incubated at 37°C (5% CO_2_, 95% relative humidity) and maintained at less than 70% to 80% confluence (approximately 75,000 cells/cm^2^). Cell heterologously expressing APJ or AT1 were not used after 10 passages. HRECs were not used in any experiments after five passages.

### HREC APJ receptor immunofluorescence labeling

HREC cells were seeded on four chamber Lab-Tek Chambered Coverglass (Thermo Scientific; 1500 cells/chamber) for cell imaging in EGM-2 medium (Lonza) in a 5% CO_2_ atmosphere at 37°C for 1 day. Chambers were washed with ice-cold PBS and fixed for 10 min with paraformaldehyde (4%) on ice then washed in PBS containing Triton X-100 (0.2% v/v) for 2 x 20 min to permeabilize the cells. To block nonspecific protein-protein interactions, cells were washed with Odyssey Blocking Buffer and PBS (1:1 v/v) containing 5% BSA for 1 h at room temperature then incubated with rabbit anti -APJ antibodies (Abcam; ab66218, ab84296, ab140508; 1:500 dilution, 2μg/ml) overnight at +4°C. Goat anti-rabbit Alexa Fluor 594 secondary IgG (H+L; Invitrogen, 1:500 dilution, 4μg/ml) and DAPI Fluoromount-G (SouthernBiotech) were used for anti-APJ receptor antibodies labeling and cell nuclei staining for confocal fluorescence microscopy detection.

### HREC APJ receptor aggregation

1×10^6^ HREC cells were labeled with 0.5 μM carboxyfluorescein succinimidyl ester (CFSE) at 37°C for 15 min in EBM-2 media. After incubation cells were centrifuged and resuspended in EGM-2 media then 1700 cells were transferred to a well of Lab-Tek Chambered coverglass and incubated in a 5% CO_2_ atmosphere at 37°C overnight. To induce APJ receptor internalization, apelin-13 (100 μM) was applied for 1.5, 3, 6, 12, 30, 60 and 240 min in separate chambers. After incubation the cells were washed, fixed, permeabilized, blocked and labeled with anti–APJ receptor antibody (ab140508, 1:500 dilution, 2 μg/ml) and secondary Alexa Fluor 594 conjugated antibody and DAPI as stated above.

### Confocal fluorescence microscopy

Immunofluorescent images (TIFF; 16 bit) were acquired by laser-scanning confocal microscopy with an A1R confocal microscope (Nikon Instruments) in single channel operation mode. Excitation lasers and emission filters were selected based on staining fluorochromes. Images were obtained with a Plan Apo 60x/1.40 oil objective (Nikon) as optical section Z-stacks. Identical acquisition settings were used for imaging for all time points using the NIS Elements AR software (Nikon). Specific fluorophore intensities were quantified on the Sum Slices Z-projection of the optical sections using ImageJ software (NIH). Area of nucleus of each cells from all time points were selected as region of interest (ROI) based on their DAPI staining.

### Cell proliferation and migration assay

The proliferation of HRECs was monitored by the crystal violet assay as described in our previous publication [87]. Briefly, 2,500 cells in 100μL Endothelial Basal Medium (EBM) (Lonza) in the presence of 50 ng/mL recombinant human VEGF165 (Biolegend, San Diego, CA, USA), were incubated in 24-well plates for 24 h followed by 48 h incubation with VEGF, Ap13 and/or the APJ antagonists. At the end of the incubation, cells were fixed in 4% paraformaldehyde in PBS for 15 min and stained in a solution of 0.1% crystal violet (Sigma Aldrich, St. Louis, MO), 10% ethanol for 5 min. After washing three times with PBS, the plates were air-dried and the remaining stain was dissolved in, 10% acetic acid and absorbance measured with a microplate reader at 540 nm.

Migration was determined by the QCM™ chemotaxis 5 μm 96-well cell migration assay (Chemicon International, Inc.) as per the manufacture’s protocol. Briefly, HREC migration in response to recombinant human VEGF165 (50 ng/mL) and/or Ap13 (10 nM) was determined alone or in the presence of the APJ antagonist AQ (10 μM). After 16 h, cells that migrated to the basal side of the chamber insert membrane were detached, lysed and detected using CyQuant GR™ dye. Migration is expressed as percent increase in migrated cells compared to untreated cells.

### *In vitro* tube formation assay

*In vitro* tube formation assays were carried out as previously described [85]. Briefly, near confluent microvascular endothelial cells were pretreated with VEGF (100 ng/ml) for 2 h and then treated for 24 h with test compounds at serial concentrations, as indicated. Cells without VEGF treatment or with VEGF only were used as control. The cells were then detached and plated sparsely (2.5 ×10^4^/well) on 24-well plates coated with 12.5% (v/v) Matrigel (BD, Franklin Lakes, NJ) and left overnight. The medium was then aspirated and 250 μl/well of 12.5% Matrigel was overlaid on the cells for 2 h to allow the polymerization of Matrigel, followed by addition of 500 μl/well of basal medium MCD131 with 10% fetal calf serum (FCS) for 24 h. The following day, the culture plates were observed under a phase contrast microscope and photographed at random in five fields (×10). The tubule length (mm/mm^2^) per microscope field was quantified.

### HTS, cAMP assay

All reagents unless otherwise specified are components of the cAMP HitHunter (DiscoveRx) kit. Ligand Buffer + 60 μM Forskolin (Cayman Chemical, Ann Arbor, MI) was made fresh on the day of the experiment and used for the dilution of positive and negative controls as well as all peptides. CHO-K1-APJ cells were dispensed into a 1536-well tissue culture microplate (Corning, Corning, NY) using a Multidrop at a seeding density of 1,000 cells/well and returned to the incubator. The next day, the cell culture media was removed and replaced with 15 μl/well of assay buffer (1X HBSS, 10 mM HEPES) containing anti-cAMP antibody (DiscoveRx). Using the Janus Automated Workstation (Perkin Elmer), 5 μl of Ap13, ligand buffer (vehicle, 1X HBSS, 10 mM HEPES, 0.1% BSA), or peptide (10-point concentrations) were added to all wells, and subsequently incubated at 37°C for 30 min. A working solution of ED/Lysis/CL substrate (20 μl/well) was added to all wells and incubated for 1 h at room temperature in the dark prior to a final addition of EA Reagent (20 μl/well). Plates were incubated at room temperature in the dark for 3 hours prior to chemiluminescence detection on an EnVision (Perkin Elmer) using a counting time of 1 s/well.

### APJ and AT1 β-arrestin recruitment assays

CHO-K1 cells engineered to over-express APJ or AT1, and β-arrestin were removed from flasks using TrypLE Select (1X), no phenol red (Life Technologies, Grand Island, NY), centrifuged, and resuspended in CP2 Reagent (DiscoveRx, Fremont, CA). Cells were counted using a Countess Automated Cell Counter (Invitrogen, Carlsbad, CA) and 5,000 cells/well were plated in a 384-well tissue culture treated microplate (Corning, Corning, NY). All plates were incubated overnight at 37°C, 5% CO2 in a final volume of 25 μl/well. Following incubation, 5 μl of peptides (10-point concentrations) and controls prepared in ligand buffer (1X HBSS, 10 mM HEPES, 0.1% BSA) were added to cells and incubated at 37°C for 1.5 h. Using a Multidrop Combi Reagent Dispenser (Thermo Scientific), 12 μl of Detection Reagent (DiscoveRx) comprised of substrate and co-factors was added to all wells and incubated at room temperature in the dark for 1 h. Chemiluminescent signal was detected on an EnVision Multi-label plate reader (Perkin Elmer) using a counting time of 1 s/well. The robustness of data from each screening plate and run was monitored by Z’ and Signal Window calculation [88].

### Radioligand competition binding assay

Prior to the initiation of the assay, soaking buffer (50 mM Tris-HCl pH 7.5, 0.5% polyethyleneimine), assay buffer (25 mM HEPES pH 7.5, 10 mM MgCl_2_, 1mM CaCl_2_, 0.5% BSA, protease inhibitor), and wash buffer (50 mM Tris-HCl pH 7.5, 0.5% BSA) were prepared. Soaking buffer (300 μl/well) was added to a 96-well GF/C filter plate (MultiScreen Harvest plate, Millipore) and left to equilibrate at room temperature for 3 h. Briefly, 25 μl peptide (8-point concentrations), 25 μl of 0.2 nM [^125^I] Glp65, Nle75, Tyr77-Ap13 (Perkin Elmer), and 150 μl APJ membrane (Perkin Elmer) diluted 1:150 in assay buffer, were added to a 96-well HB OptiPlate (Perkin Elmer) and incubated at room temperature for 45 min. Following incubation, contents were transferred from the OptiPlate to the pre-wet GF/C filter plate, and immediately underwent vacuum filtration. The filter plate was washed five times with 200 μl ice cold wash buffer and left at room temperature overnight to equilibrate. The next day, 20 μl scintillation liquid (Microscint 20, Perkin Elmer) was added and radioactivity quantified using a TopCount NXT (Perkin Elmer) microplate scintillation and luminescence counter.

Competition binding analysis for the agonist [^125^I]-Glp65, Nle75, Tyr7-Ap13 by SBI-612 in the absence and presence of Ap13 (100 nM) was performed according to the equation (GraphPad Prism version 7):

$$Y=Bottom+\frac{Top-Bottom}{{1+10}^{\left(X-Log{IC}_{50}\right)}}$$ (1)

where Y represents specific binding of the radioligand, Top is the specific binding of the radioligand in the absence of competing ligand, Bottom is the specific binding of the radioligand equivalent to nonspecific binding, IC_50_ is the concentration of competing ligand that produces radioligand binding halfway between the Top and Bottom, and X is the logarithm of the concentration of the competing ligand. The Cheng & Prusoff equation was used to convert IC_50_ estimates to equilibrium dissociation constants [89].

### Microsome stability assay

Hepatic microsomes stability assays were performed as reported before [90]. Briefly, 3 μL of 25 μM compound solution in acetonitrile were incubated with 123 μL of mouse, human or rat liver microsomes (Xenotech, Kansas City, KS). After preincubation at 37°C for 10 min, enzyme reactions were initiated by adding 120 μL of NADPH-generating system (2 mM NADP^+^, 10 mM glucose-6-phosphate, 0.4 U/ml glucose-6-phosphate dehydrogenase, and 5 mM MgCl_2_) in the presence of 100 mM potassium phosphate buffer (pH 7.4). The final concentration of each model compound used was 1 μM. The microsomal concentrations used were 1.0 mg/mL. Compounds were incubated in microsomes for 0, 5, 15, 30 and 60 min. The reactions were stopped by the addition of ice cold ACN and the reaction mixtures were centrifuged at 10,000*g* for 10 min before the supernatant was removed for analysis. 10 μL of the resulting extract were injected on a Thermo HPLC system equipped with PAL CTC plate sampler (96-well plate), Dionex Ultimate 3000 binary pump (flow rate at 0.600 mL/ min), Dionex Ultimate 3000 thermostatted column compartment (temperature at 40°C), Thermo Endura Mass Spectrometer (ESI source), using a Thermo Scientific Accucore C18 (2.6 μM, 2.1 x 50 mm) column. A gradient was run starting at 95% H_2_O (0.1% formic acid) and 5% ACN (0.1% formic acid) during the first 0.5 min, then under gradient condition of 5–100% ACN (0.1% formic acid) from minute 0.5 to 3.5, finishing at 95% H_2_O (0.1% formic acid) and 5% ACN (0.1% formic acid) over 0.5 min, with another 1 min at 95:5 to re-equilibrate. For quantification, fresh neat samples of AQ and DEAQ were solubilized in DMSO then spiked into water to generate calibration curve.

### Cytotoxicity

Fa2N-4 immortalized human hepatocytes (Xenotech) or HREC cells were grown to subconfluency in wells of a 96-well plate and exposed to different concentrations of compounds for 24 h after which the cells were washed and the media and compounds replaced. The cells were returned to the incubator for another 24 h. The extent of cell death (cell lysis) was determined by ATP-Lite reagent (Perkin-Elmer).

### Curve fit and statistical analysis

All concentration response curves were analyzed to determine EC_50_ and E_max_ using the following equation:

$$Y=\frac{100}{\left(1+10^{\left(LogEC50-X\right)}*HillSlope\right)}$$ (2)

where Y = the normalized response (0–100%), X is the log of concentration of compound tested, and the Hill slope is set equal to 1.

Except for the primary assay in the HTS campaign, all experiments were repeated at least three times. Results are expressed as mean ± SEM. The Mann-Whitney test was used to determine statistical significance of the densitometry data of Western blot analysis. Unpaired two-tailed Student’s *t*-test was performed for the significance of the results of ELISA and *in vitro* tubule formation assay. ANOVA was used to determine the significance *in vivo* CNV models using the Tukey post-hoc test for multiple comparisons. Curve fit and statistical analyses was performed using Prism 7 (GraphPad Software, Inc., La Jolla, CA) with p<0.05 considered statistically significant. T_1/2_ was calculated using the best fit curve resulting from non-linear (human) or linear (mouse and rat) regression analyses using Prism 7.

### Synthesis of aminoquinolines

The synthesis of aminoquinolines is shown in Figure F in S1 File. 4,7-dichloroquinoline (101 mg, 0.51 mmol) and ethyl-4-aminobenzoate (87 mg, 0.53 mmol) were heated in ethanol at 80°C 45 minutes then stirred at room temperature. The solids were filtered to provide ethyl 4-((7-chloroquinolin-4-yl)amino)benzoate hydrochloride (147 mg, 79%). The ester (143 mg, 0.39 mmol) was hydrolyzed with lithium hydroxide (32 mg, 1.34 mmol) in water (1 mL) and THF (4 mL) at room temperature. The mixture was partitioned with ethyl acetate and water. The aqueous phase was acidified with conc. HCl to precipitate 4-((7-chloroquinolin-4-yl)amino)benzoic acid hydrochloride (131 mg, 99%). The acid (20 mg, 0.06 mmol) was activated with HATU (30 mg, 0.08 mmol) and triethylamine (0.045 mL, 0.32 mmol) in THF for 30 minutes prior to the introduction of excess ammonia (0.24 mL, 0.5 M in THF, 0.12 mmol). After stirring overnight the mixture was diluted with water, treated with sodium bicarbonate, and the product was extracted with ethyl acetate. The crude material was purified by reverse phase HPLC to provide 4-((7-chloroquinolin-4-yl)amino)benzamide (16 mg, 90%). Proton NMR spectra for synthesized analogs are shown in Figures G—J in S1 File.

## Supporting information

S1 File Supporting information for the anti-malarial drug, amodiaquine, is an apelin-receptor antagonist that blocks angiogenesis in vitro and in vivo. **(DOCX).** Table A. Results of quantitative PCR showing the endogenous mRNA levels of APJ in HRECs compared to heterologously expressed APJ in CHO-K1 cells and parental cells lacking APJ. Results presented as relative expression level of APJ normalized to endogenous calibrator target (GAPDH), and as a relative percent expression normalized to the CHO-K1 cells heterologously overexpressing human APJ. **Figure A. Human retinal endothelial cells (HREC) express APJ. (A, B, C)** APJ protein was detected and visualized in HRECs by immunocytochemistry using the anti-APJ antibodies indicated, and an Alexa488 conjugated secondary antibody. **(D)** A control experiment in which the primary anti-APJ antibody was omitted shows the specificity of these antibodies for APJ. **Figure B. Ap13 does not synergize with VEGF to induce HREC tube formation.** HREC cells were exposed to both Ap13 alone (black bars) and in combination with VEGF (10 ng/mL, grey bars). Increasing concentrations of Ap13 up to 100 nM had no observable synergistic effect with VEGF compared to AP13 alone. There was no statistically significant difference between either treatment (p > 0.5, by Student’s t-test). **Figure C. ML221 blocks VEGF-induced HREC tube formation.** Data plotted is the mean ± SEM length of endothelial tubes measured in micrometers (μm), normalized to vehicle control. Mean and SEM are calculated from an experiment that was performed twice with each treatment condition tested in triplicate (*n* = 3). NS = not significant; ** = p<0.01; *** = p<0.001 vs vehicle; † = p<0.0001 compared to cells incubated with VEGF alone (100 ng/mL) as determined by ANOVA with Tukey’s multiple comparison test. **Figure D. Metabolism of AQ to DEAQ by hepatic microsomes.** The conversion of AQ to the metabolite desethylaminoquinoline (DEAQ) was monitored *in vitro* using (**A**) mouse, (**B**) human and (**C**) rat hepatic microsomes. The consumption of AQ and a production of DEAQ was measured by quantitative LC-MS/MS using internal standards and a standard curve for both AQ and DEAQ. Data points represent the mean ± SEM ng/mL of each compound from an experiment performed in duplicate. Curves represent the best fit non-linear regression analysis for AQ and linear regression analysis for DEAQ as described in materials and methods, using GraphPad Prsim7. **Figure E. Concentration response of DEAQ, the primary human metabolite of AQ, at APJ.** Data are mean ± SEM (n = 3). Curve represents the best fit non-linear regression analysis calculated using a 4-paramter logistic with GraphPad Prism7. **Figure F. Synthetic scheme depicting the facile synthesis of aminoquinolines used in this study.** Conditions: i) ethyl-4-aminobenzoate, EtOH, 80°C; ii) LiOH, H_2_O, THF; iii) HATU, NH_3_, Et_3_N. **Figure G. Proton NMR spectra for 1. 4-((7-chloroquinolin-4-yl)amino)benzamide.** ^1^H NMR (500 MHz, DMSO-*d*_6_) δ 9.28 (s, 1H), 8.56 (d, *J* = 5.2 Hz, 1H), 8.41 (d, *J* = 9.0 Hz, 1H), 7.95–7.88 (m, 3H), 7.61 (dd, *J* = 9.0, 2.2 Hz, 1H), 7.41 (d, *J* = 8.6 Hz, 2H), 7.26 (s, 1H), 7.15 (d, *J* = 5.3 Hz, 1H). LRMS (ESI+ve): Calculated for C_16_H_12_ClN_3_O, [M+H] = 298.07, observed [M+H] = 298.21. **Figure H. Proton NMR spectra for 4. 7-chloro-N-(4-methoxyphenyl)quinolin-4-amine.** ^1^H NMR (500 MHz, DMSO-*d*_6_) δ 8.96 (s, 1H), 8.42 (d, *J* = 9.1 Hz, 1H), 8.39 (d, *J* = 5.4 Hz, 1H), 7.86 (d, *J* = 2.2 Hz, 1H), 7.54 (dd, *J* = 9.0, 2.3 Hz, 1H), 7.28 (d, *J* = 8.8 Hz, 2H), 7.02 (d, *J* = 8.8 Hz, 2H), 6.62 (d, *J* = 5.4 Hz, 1H), 3.79 (s, 3H). LRMS (ESI+ve): Calculated for C_16_H_13_ClN_2_O, [M+H] = 285.08, observed [M+H] = 285.22. **Figure I. Proton NMR spectra for 5. 2-((7-chloroquinolin-4-yl)amino)benzoic acid.** ^1^H NMR (500 MHz, DMSO-*d*_6_) δ 8.63 (d, *J* = 9.1 Hz, 1H), 8.53 (d, *J* = 6.7 Hz, 1H), 8.10 (d, *J* = 8.4 Hz, 2H), 7.88 (d, *J* = 8.9 Hz, 1H), 7.78 (t, *J* = 7.6 Hz, 1H), 7.64 (d, *J* = 7.9 Hz, 1H), 7.52 (t, *J* = 7.6 Hz, 1H), 6.72 (d, *J* = 6.6 Hz, 1H). LRMS (ESI+ve): Calculated for C_16_H_11_ClN_2_O_2_, [M+H] = 299.06, observed [M+H] = 299.19. **Figure J. Proton NMR for 6. (2-((7-chloroquinolin-4-yl)amino)phenyl)(morpholino) methanone.** ^1^H NMR (500 MHz, Chloroform-*d*) δ 8.54 (d, *J* = 5.3 Hz, 1H), 7.96 (d, *J* = 2.1 Hz, 1H), 7.85 (d, *J* = 9.0 Hz, 1H), 7.62 (dd, *J* = 8.2, 1.2 Hz, 1H), 7.42 (dd, *J* = 8.9, 2.2 Hz, 1H), 7.38 (ddd, *J* = 8.4, 7.4, 1.6 Hz, 1H), 7.26 (dd, *J* = 7.7, 1.6 Hz, 1H), 7.10 (d, *J* = 5.3 Hz, 1H), 7.06 (td, *J* = 7.6, 1.1 Hz, 1H), 3.58 (s, 8H). LRMS (ESI+ve): Calculated for C_20_H_18_ClN_3_O_2_, [M+H] = 368.12, observed [M+H] = 368.32.
(DOCX)
