# Supplementary material for: Repurposing antimalarial aminoquinolines and related compounds for treatment of retinal neovascularization
Source: PLoS One. 2018 Sep 12;13(9):e0202436. doi: 10.1371/journal.pone.0202436 (PMC6135396; doi:10.1371/journal.pone.0202436)

**S1. Supporting information for the anti-malarial drug, amodiaquine, is an apelin-receptor antagonist that blocks angiogenesis in vitro and in vivo.**

**Repurposing antimalarial aminoquinolines and related compounds for treatment of retinal neovascularization**

Danielle McAnally^1,2^, Khandaker Siddiquee^1^, Ahmed Gomaa^4^, Andras Szabo^1^, Stefan Vasile^2^, Patrick R. Maloney^2^, Daniela B. Divlianska^2^, Satyamaheshwar Peddibhotla^2^ , Camil J. Morfa^2^, Paul Hershberger^2^, Rebecca Falter^2^, Robert Williamson^2^, David B. Terry^2^, Rafal Farjo,^5^ Anthony B. Pinkerton^3^, Xiaping Qi^4,6^, Judith Quigley^4^, Michael E. Boulton^4,6^, Maria B. Grant ^4,6^, and Layton H. Smith^1,2^ *

^1^ Cardiovascular Pathobiology Program, Diabetes and Obesity Research Center, Sanford Burnham Prebys Medical Discovery Institute, Orlando, Florida, United States of America

^2^ Conrad Prebys Center for Chemical Genomics, Sanford Burnham Prebys Medical Discovery Institute, Orlando, Florida, United States of America

^3^ Conrad Prebys Center for Chemical Genomics, Sanford Burnham Prebys Medical Discovery Institute, La Jolla, California, United States of America

^4^ Department of Ophthalmology, Indiana University School of Medicine Indianapolis, Indiana, United States of America

^5^ EyeCRO LLC, Oklahoma City, Oklahoma, United States of America

^6^ Department of Ophthalmology, University of Alabama, Birmingham, Alabama, United States of America

*Corresponding author

E-mail: lhsmith@sbpdiscovery.org (L.H.S.)

**Supporting Information**

**Materials and Methods**

**Quantitative Real-Time Reverse-Transcription Polymerase Chain Reaction**

Total RNA was extracted using Trizol reagent (Invitrogen, Carlsbad, CA) and RNeasy Mini Kit (Qiagen, Valencia, CA) and made into cDNA using SuperScript III (Invitrogen). qPCR was performed on the Realplex4 Real Time PCR Detection System (Eppendorf, Westbury, NY) using TaqMan probe (Applied Biosystems, Foster City, CA) sets (human APLNR (Hs00270873_s1 ) and GAPDH (Hs02758991_g1 )). Experimental cycle threshold (Ct) values were normalized to β-actin or GAPDH measured on the same plate, and fold differences in gene expression were determined using the 2−ΔΔCt method [28].

**Table A. Results of quantitative PCR showing the endogenous mRNA levels of APJ in HRECs compared to heterologously expressed APJ in CHO-K1 cells and parental cells lacking APJ.** Results presented as relative expression level of APJ normalized to endogenous calibrator target (GAPDH), and as a relative percent expression normalized to the CHO-K1 cells heterologously overexpressing human APJ.

| **Cell type** | **Average C*t* Values** | **Relative Expression**  **(2^-△C^*^t^*)** | **Relative Expression**  **(% CHO-K1-APJ)** |
| --- | --- | --- | --- |
| **CHO-K1** | >40 | ND | ND |
| **CHOK-K1-APJ** | 20.37 ± 0.32 | 1.20 ± 0.32 | 100 |
| **HREC** | 25.83 ± 0.03 | 8.37 ± 0.03 | 0.73 ± 0.16 |

**Figure A. Human retinal endothelial cells (HREC) express APJ. (A, B, C)** APJ protein was detected and visualized in HRECs by immunocytochemistry using the anti-APJ antibodies indicated, and an Alexa488 conjugated secondary antibody. **(D)** A control experiment in which the primary anti-APJ antibody was omitted shows the specificity of these antibodies for APJ.


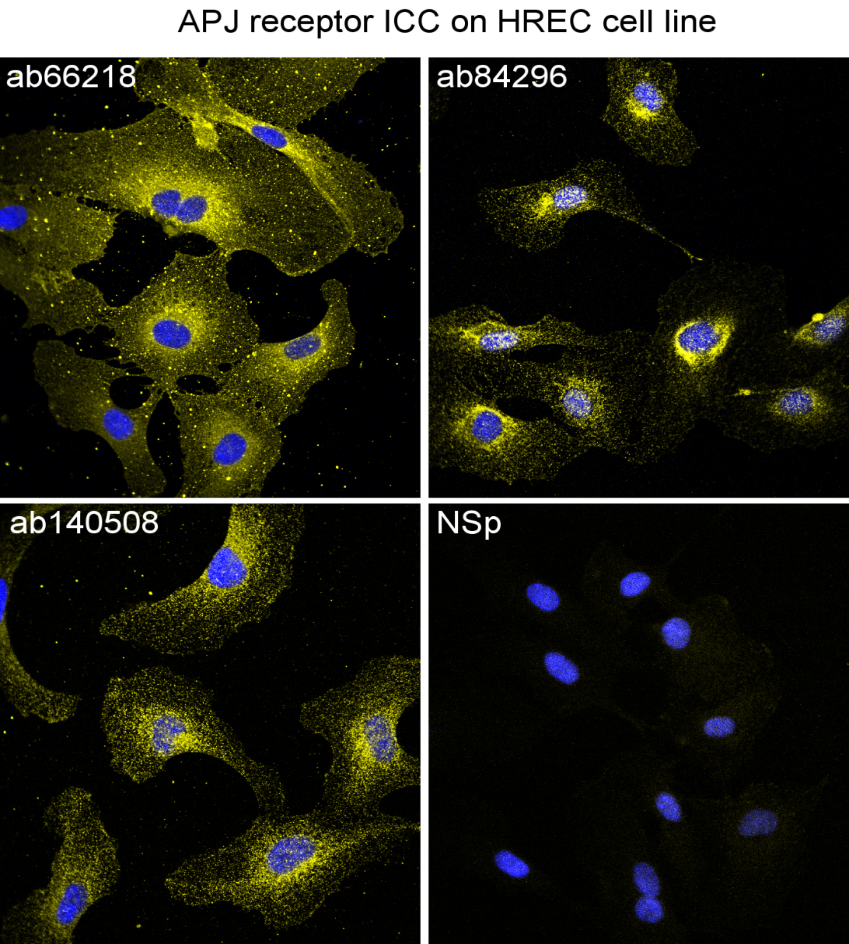


**A**

**B**

**C**

**D**

**Figure B. Ap13 does not synergize with VEGF to induce HREC tube formation.** HREC cells were exposed to both Ap13 alone (black bars) and in combination with VEGF (10 ng/mL, grey bars). Increasing concentrations of Ap13 up to 100 nM had no observable synergistic effect with VEGF compared to AP13 alone. There was no statistically significant difference between either treatment (p > 0.5, by Student’s t-test)

**Figure C. ML221 blocks VEGF-induced HREC tube formation.** Data plotted is the mean ± SEM length of endothelial tubes measured in micrometers (µm), normalized to vehicle control. Mean and SEM are calculated from an experiment that was performed twice with each treatment condition tested in triplicate (*n* = 3). NS = not significant; ** = p<0.01; *** = p<0.001 vs vehicle; ✝ = p<0.0001 compared to cells incubated with VEGF alone (100 ng/mL) as determined by ANOVA with Tukey’s multiple comparison test.


**Figure D. Metabolism of AQ to DEAQ by hepatic microsomes.** The conversion of AQ to the metabolite desethylaminoquinoline (DEAQ) was monitored *in vitro* using (**A**) mouse, (**B**) human and (**C**) rat hepatic microsomes. The consumption of AQ and a production of DEAQ was measured by quantitative LC-MS/MS using internal standards and a standard curve for both AQ and DEAQ. Data points represent the mean ± SEM ng/mL of each compound from an experiment performed in duplicate. Curves represent the best fit non-linear regression analysis for AQ and linear regression analysis for DEAQ as described in materials and methods, using GraphPad Prsim7.

**Figure E. Concentration response of DEAQ, the primary human metabolite of AQ, at APJ.** Data are mean ± SEM (n = 3). Curve represents the best fit non-linear regression analysis calculated using a 4-paramter logistic with GraphPad Prism7.

**Figure F. Synthetic scheme depicting the facile synthesis of aminoquinolines used in this study.** Conditions: i) ethyl-4-aminobenzoate, EtOH, 80°C; ii) LiOH, H_2_O, THF; iii) HATU, NH_3_, Et_3_N.

**Figure G. Proton NMR spectra for 1. 4-((7-chloroquinolin-4-yl)amino)benzamide.** ^1^H NMR (500 MHz, DMSO-*d*_6_) δ 9.28 (s, 1H), 8.56 (d, *J* = 5.2 Hz, 1H), 8.41 (d, *J* = 9.0 Hz, 1H), 7.95 – 7.88 (m, 3H), 7.61 (dd, *J* = 9.0, 2.2 Hz, 1H), 7.41 (d, *J* = 8.6 Hz, 2H), 7.26 (s, 1H), 7.15 (d, *J* = 5.3 Hz, 1H). LRMS (ESI+ve): Calculated for C_16_H_12_ClN_3_O, [M+H] = 298.07, observed [M+H] = 298.21.


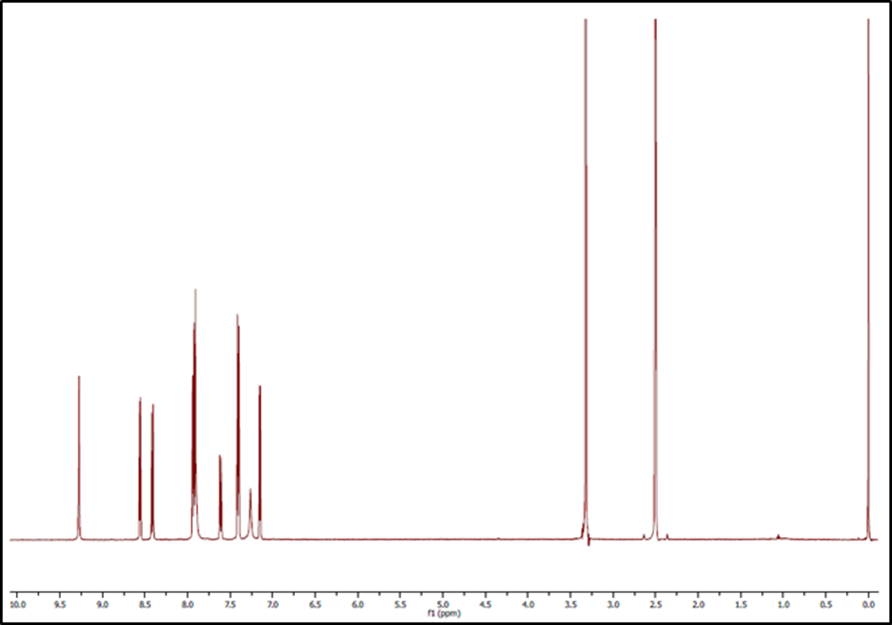


**Figure H. Proton NMR spectra for 4. 7-chloro-N-(4-methoxyphenyl)quinolin-4-amine.** ^1^H NMR (500 MHz, DMSO-*d*_6_) δ 8.96 (s, 1H), 8.42 (d, *J* = 9.1 Hz, 1H), 8.39 (d, *J* = 5.4 Hz, 1H), 7.86 (d, *J* = 2.2 Hz, 1H), 7.54 (dd, *J* = 9.0, 2.3 Hz, 1H), 7.28 (d, *J* = 8.8 Hz, 2H), 7.02 (d, *J* = 8.8 Hz, 2H), 6.62 (d, *J* = 5.4 Hz, 1H), 3.79 (s, 3H). LRMS (ESI+ve): Calculated for C_16_H_13_ClN_2_O, [M+H] = 285.08, observed [M+H] = 285.22.


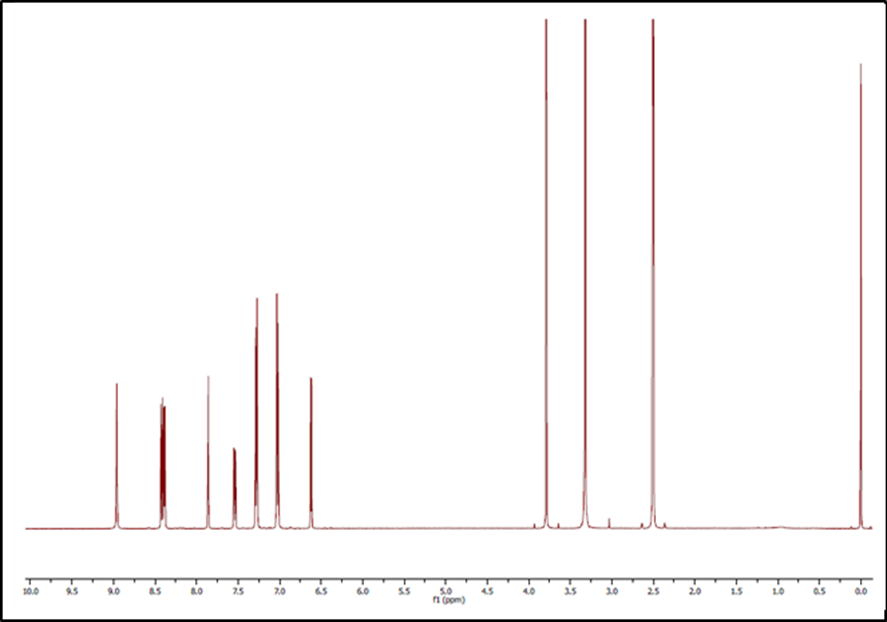


**Figure I.** **Proton NMR spectra for 5. 2-((7-chloroquinolin-4-yl)amino)benzoic acid.** ^1^H NMR (500 MHz, DMSO-*d*_6_) δ 8.63 (d, *J* = 9.1 Hz, 1H), 8.53 (d, *J* = 6.7 Hz, 1H), 8.10 (d, *J* = 8.4 Hz, 2H), 7.88 (d, *J* = 8.9 Hz, 1H), 7.78 (t, *J* = 7.6 Hz, 1H), 7.64 (d, *J* = 7.9 Hz, 1H), 7.52 (t, *J* = 7.6 Hz, 1H), 6.72 (d, *J* = 6.6 Hz, 1H). LRMS (ESI+ve): Calculated for C_16_H_11_ClN_2_O_2_, [M+H] = 299.06, observed [M+H] = 299.19.


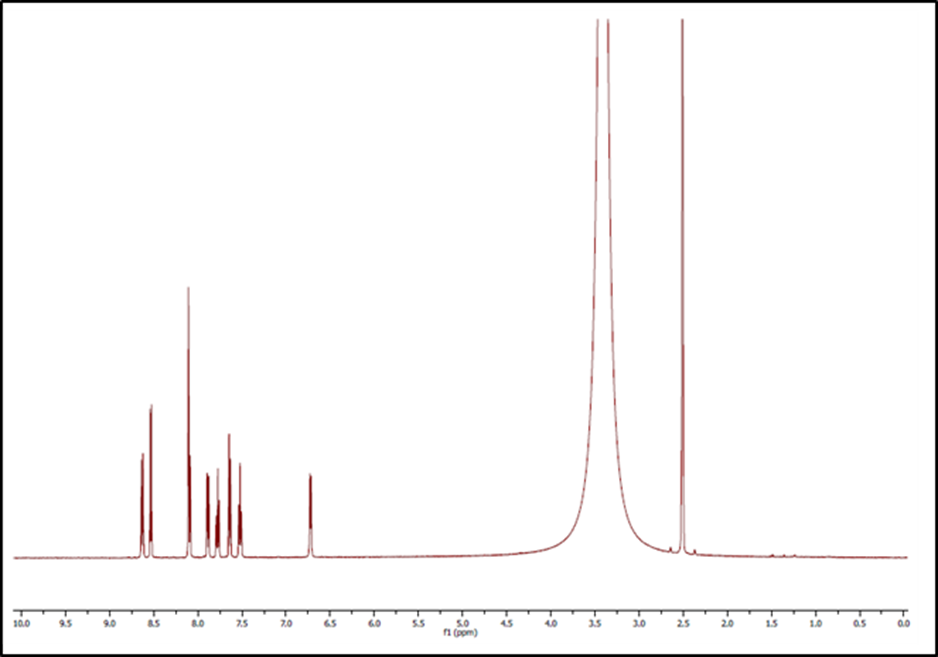


**Figure J.** **Proton NMR for 6. (2-((7-chloroquinolin-4-yl)amino)phenyl)(morpholino) methanone.**  ^1^H NMR (500 MHz, Chloroform-*d*) δ 8.54 (d, *J* = 5.3 Hz, 1H), 7.96 (d, *J* = 2.1 Hz, 1H), 7.85 (d, *J* = 9.0 Hz, 1H), 7.62 (dd, *J* = 8.2, 1.2 Hz, 1H), 7.42 (dd, *J* = 8.9, 2.2 Hz, 1H), 7.38 (ddd, *J* = 8.4, 7.4, 1.6 Hz, 1H), 7.26 (dd, *J* = 7.7, 1.6 Hz, 1H), 7.10 (d, *J* = 5.3 Hz, 1H), 7.06 (td, *J* = 7.6, 1.1 Hz, 1H), 3.58 (s, 8H). LRMS (ESI+ve): Calculated for C_20_H_18_ClN_3_O_2_, [M+H] = 368.12, observed [M+H] = 368.32.


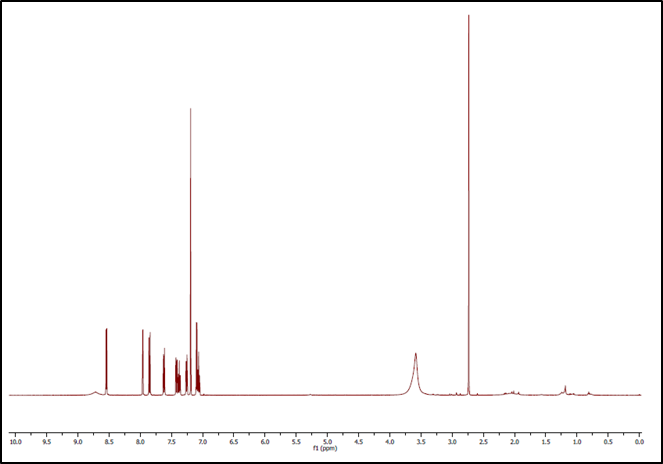

Supplement: S1 File — (DOCX). Table A. Results of quantitative PCR showing the endogenous mRNA levels of APJ in HRECs compared to heterologously expressed APJ in CHO-K1 cells and parental cells lacking APJ. Results presented as relative expression level of APJ normalized to endogenous calibrator target (GAPDH), and as a relative percent expression normalized to the CHO-K1 cells heterologously overexpressing human APJ. Figure A. Human retinal endothelial cells (HREC) express APJ. (A, B, C) APJ protein was detected and visualized in HRECs by immunocytochemistry using the anti-APJ antibodies indicated, and an Alexa488 conjugated secondary antibody. (D) A control experiment in which the primary anti-APJ antibody was omitted shows the specificity of these antibodies for APJ. Figure B. Ap13 does not synergize with VEGF to induce HREC tube formation. HREC cells were exposed to both Ap13 alone (black bars) and in combination with VEGF (10 ng/mL, grey bars). Increasing concentrations of Ap13 up to 100 nM had no observable synergistic effect with VEGF compared to AP13 alone. There was no statistically significant difference between either treatment (p > 0.5, by Student’s t-test). Figure C. ML221 blocks VEGF-induced HREC tube formation. Data plotted is the mean ± SEM length of endothelial tubes measured in micrometers (μm), normalized to vehicle control. Mean and SEM are calculated from an experiment that was performed twice with each treatment condition tested in triplicate (n = 3). NS = not significant; ** = p<0.01; *** = p<0.001 vs vehicle; † = p<0.0001 compared to cells incubated with VEGF alone (100 ng/mL) as determined by ANOVA with Tukey’s multiple comparison test. Figure D. Metabolism of AQ to DEAQ by hepatic microsomes. The conversion of AQ to the metabolite desethylaminoquinoline (DEAQ) was monitored in vitro using (A) mouse, (B) human and (C) rat hepatic microsomes. The consumption of AQ and a production of DEAQ was measured by quantitative LC-MS/MS using internal standards and a standar [file pone.0202436.s001.docx]
